# Adversarial-Aware Deep Learning System Based on a Secondary Classical Machine Learning Verification Approach

**DOI:** 10.3390/s23146287

**Published:** 2023-07-11

**Authors:** Mohammed Alkhowaiter, Hisham Kholidy, Mnassar A. Alyami, Abdulmajeed Alghamdi, Cliff Zou

**Affiliations:** 1 College of Engineering and Computer Science, University of Central Florida, Orlando, FL 32816, USA; mnassar.alyami@knights.ucf.edu (M.A.A.); a.alghamdi@knights.ucf.edu (A.A.); 2 College of Computer Engineering and Science, Prince Sattam Bin Abdulaziz University, Al-Kharj 11942, Saudi Arabia; 3 College of Engineering, SUNY Polytechnic Institute, Utica, NY 13502, USA; kholidh@sunypoly.edu

**Keywords:** computer security, deep neural networks, image forensics, adversarial machine learning, image manipulation detection

## Abstract

Deep learning models have been used in creating various effective image classification applications. However, they are vulnerable to adversarial attacks that seek to misguide the models into predicting incorrect classes. Our study of major adversarial attack models shows that they all specifically target and exploit the neural networking structures in their designs. This understanding led us to develop a hypothesis that most classical machine learning models, such as random forest (RF), are immune to adversarial attack models because they do not rely on neural network design at all. Our experimental study of classical machine learning models against popular adversarial attacks supports this hypothesis. Based on this hypothesis, we propose a new adversarial-aware deep learning system by using a classical machine learning model as the secondary verification system to complement the primary deep learning model in image classification. Although the secondary classical machine learning model has less accurate output, it is only used for verification purposes, which does not impact the output accuracy of the primary deep learning model, and, at the same time, can effectively detect an adversarial attack when a clear mismatch occurs. Our experiments based on the CIFAR-100 dataset show that our proposed approach outperforms current state-of-the-art adversarial defense systems.

## 1. Introduction

Machine learning (ML) is a type of artificial intelligence (AI) that focuses on creating algorithms and models by enabling computers to learn and make predictions or decisions without explicit programming. However, as ML technology, especially deep learning, continues to advance in computer vision, the risk of adversarial attacks is becoming more prevalent. Adversarial attacks involve image manipulation designed to deceive computer vision tasks, making the image appear correct to human perception [1]. These attacks can lead to harmful failures in sensitive computer-vision-based applications, such as autonomous vehicles misinterpreting a STOP sign as a SPEED LIMIT 65 sign. The demand for AI applications is increasing, which may increase the risk of these attacks if the technology is not secured before it is marketed. Therefore, researchers have been developing algorithms and systems to prevent adversarial attacks. This paper presents a novel adversarial-aware deep learning system that uses a classical ML algorithm as an auxiliary verification approach.

Deep neural network (DNN) theory, also called deep learning, accelerates the development of computer vision applications to advance the work presented in  [2,3,4,5,6]. Unlike other ML approaches, DNNs can quickly learn complex patterns and representations from large and high-dimensional datasets. Therefore, according to a study by Stone [7], DNN technology is expected to be used in an expanding range of real-world applications within the next decade. Examples of these applications include autonomous vehicles, security surveillance cameras, and health care. However, this technology faces serious security challenges because of two factors. One is the high dimensionality and complexity of the input data to DNN models, which means that it is difficult to catch all potential attacks, as adversarial attackers can insert small but sufficient perturbations to mislead the system. Second is the non-linearity in the decision boundaries of DNNs, resulting in unexpected and complex behaviors that are difficult to predict.

### 1.1. Inspiration

In this paper, our proposed new idea in defending against adversarial attacks is inspired by analyzing communication war in the real world, as described below. Suppose a war scenario or simulation in which the Blue team uses satellite communication to operate its military. If the other side, the Red team, is somehow capable of modifying the Blue team’s satellite communication without being detected, then the Blue team is misled and could lose the war eventually. In defending against such an attack in disruption of its communication, the Blue team could add a secondary radiotelegraphy system to complement its main satellite communication because radiotelegraphy, relying on a completely different mechanism, cannot be disrupted by the Red team’s satellite attack methodology. Although radiotelegraphy using Morse code has very limited bandwidth, it can transmit summary data that matches the complete data transmitted via satellite communication. In this system, if the receiver finds out that the information between the radiotelegraphy and the satellite communication does not match, it can tell that a Red team satellite-based attack is ongoing and will therefore not be fooled by the misinformation.

Our proposed defense system against adversarial attacks uses the same philosophy as the war scenario described above. The deep learning image classification system is an analogy to satellite communication and can be compromised by various adversarial attacks. However, we propose the use of a traditional ML algorithm, such as RF, in analogy to radiotelegraphy, as the secondary verification system. Although it is less accurate than a normal deep learning image classification system, it is immune to most known adversarial attacks because it does not rely on a neural network structure. In this way, we can detect adversarial attacks easily when there is a mismatch between the outputs of the primary deep learning module and the secondary RF module.

### 1.2. Research Contributions and Paper Outline

Based on a large-scale experiment and investigation, we find a ground similarity between various adversarial attacks on different deep learning models, which motivated us to develop this research work, as illustrated in Section 2.1. The main contribution of this paper is integrating the primary deep learning model with an auxiliary traditional ML model that is not based on neural network architecture (presented in Section 2). Additionally, a new defense metric for selecting the highest Top_k predicted class probabilities of an input sample is introduced in Section 2.3. The misclassification issue of DNN models is also addressed in the same section, and an overall DNN model with improved accuracy is discussed.

Our method surpasses all other state-of-art defense methods in detecting multiple adversarial attacks using the CIFAR-100 dataset [8], which is shown in Section 3. A thorough discussion of our research is presented in Section 4, covering the solutions, the challenges, and the limitations encountered. Finally, a comprehensive conclusion is reached in Section 5, and potential future research avenues are identified to further improve the reliability of adversarial detection models.

### 1.3. Related Work

In this section, we briefly review state-of-the-art existing works on adversarial attacks and defenses. We also study the competitive detector methods that we compare our work with. These models are DkNN [9], LID [10], Mahalanibis [11], and NNIF [12].

#### 1.3.1. Adversarial Attacks

In the past few years, many adversarial attacks have been proposed; the most common attack proposed by [1] is called the fast gradient sign method (FGSM). This attack adds a small perturbation to the target image in the direction of the gradient of the loss function with respect to the human-perception content in order to misclassify the trained targeted model. It is a white-box attack where the attacker fully knows the deep learning model, including its architecture, parameters, and training data. Later, a more efficient attack known as Deepfool [13] finds the smallest perturbation necessary to cause a DNN to misclassify an input image, which increases the attack success rate compared to FGSM.

The potential of deceiving DNN models increased significantly over the past few years of adversarial attack development. Today, imperceptible perturbations can be added to input images with the flexibility of adjusting the attack goal to either a white box or a black box, such as the one proposed in [14] and named after its founders, Carlini and Wagner (CW) attack. This attack uses an optimization algorithm in order to find the smallest perturbation that minimizes a loss function that balances the size of the perturbation with the misclassification success rate. Moreover, the attack has the ability to incorporate constraints on the perturbation, such as by limiting the magnitude of the perturbation or restricting the pixel values of the perturbed image. The power of this attack raises the challenge of defense solutions against multiple attacks at once.

The white-box approach becomes more desirable for adversaries, as it was introduced by [15] and is known as the complete white-box adversary. Researchers found that the projected gradient descent (PGD) can lift any constraints on the amount of time and effort the attacker can put into finding the best attack. The iterative feature of the PGD attack makes it more effective than other attacks, such as FGSM, in finding imperceptible adversarial examples. The variety and effectiveness of adversarial attacks open a wide range of areas for researchers to develop different attacks, such as in [16,17], and to find defense mechanisms on the other side.

#### 1.3.2. Adversarial Defenses

The authors of [18] categorized the adversarial defense mechanisms in computer vision into three approaches. The first approach targets the deep learning model by making modifications to the model itself in order to make it more resistant to adversarial attacks. This approach was initially employed by researchers Szegedy and Goodfellow [1,19] in 2013 and 2014, respectively. Years later, Madry [15] delved deeper into this approach by studying the robustness of neural networks against adversarial attacks from a theoretical standpoint, using robust optimization techniques. Despite its limitations, as discussed in [15], adversarial training has garnered considerable attention from the research community. In [20], a new defense algorithm called Misclassification Aware adveRsarial Training (MART) was proposed. It distinguishes between misclassified and correctly classified examples during the training process. In another study [21], researchers suggested using dropout scheduling to enhance the efficiency of adversarial training when employing single-step methods. The authors of [22] proposed a self-supervised adversarial training method, while the authors of [23] analyzed adversarial training for self-supervision by incorporating it into pretraining.

The second approach is a defense that targets the inputs to the model by cleaning inputs to make them benign for the target model. Ref. [24] proposed ComDefend, which consists of a compression convolutional neural network (ComCNN) and a reconstruction convolutional neural network (RecCNN). The ComCNN model compresses the input image to maintain the original image structure information and purify any added perturbation. The RecCNN model, on the other hand, reconstructs the output of ComCNN to a high quality. This approach achieved high accuracy in defending against multiple adversarial attacks. GAN architecture is another technique of input transformation introduced by [25]. Their method, Defense-GAN, learns the distribution of clean images. In other words, it generates an output image close to the input image without containing the potential adversarial perturbation.

The third approach is a defense involving the addition of external modules (mainly detectors) to the target model. Among adversarial defense/detection techniques, [9] inserted a K-nearest neighbors model (*k*-NN) at every layer of the pretrained DNN model to estimate better prediction, confidence, and credibility for a given test sample. Afterward, a calibration dataset was used to compute the non-conformity of every test sample for a specific label (j). This involved counting the number of nearest neighbors along the DNN layer that differed from the chosen label (j). The researchers discovered that in cases in which an adversarial attack was launched on a test sample, the true label exhibited less similarity with the *k*-NN labels derived from the DNN activations across the layers.

The research in [10] characterized the properties of regions named adversarial subspaces by focusing on the dimensional properties using the local intrinsic dimensionality (LID). The LID method evaluates the space-filling capability of the area around a reference by analyzing the distance between the sample and its neighboring points. A classifier was trained using a dataset comprising three types of examples: adversarial as a positive class and normal and noisy (non-adversarial) as a negative class. The features of each sample associated with each category were then constructed using the LID score calculated at every DNN layer. Finally, a logistic regression (LR) model was fitted on the LID features for the adversarial detection task.

Researchers in [11] developed generative classifiers that could detect adversarial examples by utilizing DNN activations from every layer of the training set. They used a confidence score that relied on Mahalanobis distance. First, they found the mean and covariance of activations for each class and layer. Then, they measured the Mahalanobis distance between a test sample and its nearest class-conditional Gaussian using Gaussian distributions. These distances served as features to train a logistic regression classifier. The authors found that, compared to using the Euclidean distance employed in [10], the Mahalanobis distance was significantly more effective in detecting adversarial examples and resulted in improved detection results.

In a study by Cohen et al. [12], the authors utilized an influence function to create an external adversarial detector. This function calculates how much of each training sample affects the validation data, resulting in sample influence scores. Using these scores, they identified the most supportive training instances for the validation samples. To compute a ranking of the supportive training samples, a *k*-NN model is also fitted on the model activations. According to their claims, supportive samples are highly correlated with the nearest neighbors of clean test samples, whereas weak correlations were found for adversarial inputs.

## 2. Materials and Methods

This section introduces our proposed detection method in depth. It starts with the motivation, which sheds light on our research ideas. Then, we introduce our model in detail. After that, we present the adaptive design for our defense method based on application-specific security goals.

### 2.1. Motivation and Threat Model

Motivation: After multiple assessments of the different adversarial attacks on different DNN models, we notice that once the attack succeeds on one deep learning model, it succeeds on other models as well, as shown in Table 1, which was obtained by running multiple adversarial attacks (FGSM, Deepfool, CW, and PGD) on ResNet-34 [26] as a target model using the CIFAR-100 dataset. The generated adversarial samples are then tested on VGG16 [27] and DenseNet [28] DNN model classifications. We find that the accuracy of the targeted model is similar to that of untargeted DNN models. Researchers in [29] addressed the same issue, naming it “transferability“ of adversarial examples, meaning that the generated samples from adversarial attacks on one targeted DNN model may work on different untargeted DNN models. Therefore, a model obtained by a different approach is interesting to study, and we selected a random forest (RF) [30] decision-tree-based classifier model for our study, considering all the challenges of using this limited model for image classification.

Threat model: Our threat model assumes that the attacker knows there is a detection method employed but does not know what it is. In this setting, only the DNN model and its parameters are known to the adversary.

### 2.2. Proposed Methodology

**Figure 1:**
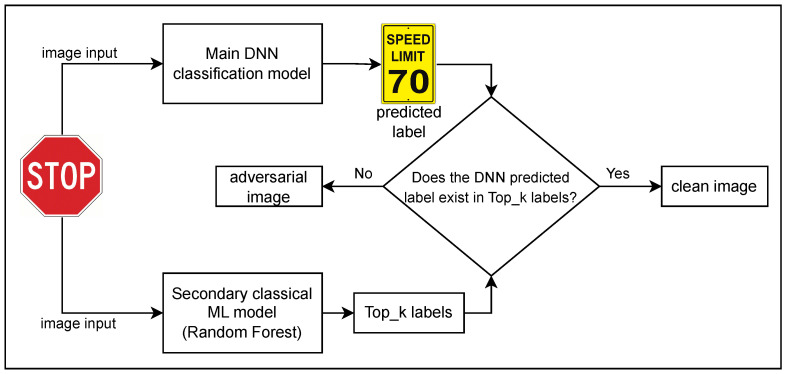
Proposed adversarial detection system design, which is composed of a primary DNN classification decision model and a secondary classical ML model for adversarial attack detection and verification.

We introduce our proposed adversarial attack detection method in this section. Our primary image classification system, shown in Figure 1, is based on the DNN approach, and we choose ResNet with 34 layers here for our investigation. The primary model could be any other DNN model that uses backpropagation because adversarial attacks exploit backpropagation to optimize the perturbations introduced to the input data on DNN models. The input is an image that could be a real image with no alteration or an adversarial generated sample from one of four attacks: FGSM, Deepfool, CW, or PGD. Our output of ResNet-34 is the highest probability index that indicates the class the image belongs to, which is referred to as Top_1 classification.

Unlike the primary approach, we use the classical ML model, the random forest (RF) model, as a secondary model for adversarial attack detection. The model can be vulnerable to adversarial attacks, as seen in [31], with the aim of deceiving the intrusion detection system. Nonetheless, we opt to use this as a secondary model because it employs a different method and does not rely on the gradient technique utilized in computer vision adversarial attacks. Therefore, the perturbation added to the images does not impact the model’s image features or the model classification performance.

**Figure 2:**
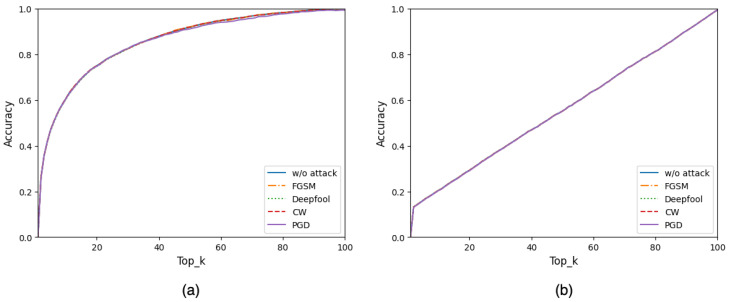
Classification accuracy over Top_k before and after different adversarial attacks using the CIFAR-100 dataset by two classical ML models: (**a**) random forest model and (**b**) the *k*-NN model. The accuracies under different adversarial attacks are almost identical; thus, those resulting curves override each other and make a single purple-colored curve.

The RF model is a decision tree module based used in regression and multiclassification problems [32,33,34,35,36]. It is an extension of the bagging method, as it utilizes both bagging and random feature selection to create an uncorrelated forest of decision trees. It also reduces overfitting and increases the diversity of the trees in the forest. The randomness in selecting the features for each tree determines and eliminates the inserted perturbations information on the adversarial samples, as illustrated in Figure 2, where the accuracies of RF model before and after different attacks are almost identical. In the same figure, the *k*-NN model is demonstrated as a classical ML model that is not affected by the added perturbations as well.

Our outputs of RF are the top k indices (Top_k) of the predicted class probabilities for the inputs. We selected Top_k and relied on it for our study to match the accuracy of the RF model with the selected DNN model on the CIFAR-100 dataset, which has 100 classes. Top_1 in the RF represents the worst accuracy, as illustrated in Figure 2, whereas Top_100 represents 100 percent accuracy because its decision is always correct, where the decision group includes all possible classes. When the *k* parameter in Top_k equals 22, the accuracy reaches around 77 percent, the same percentage as the primary DNN method prediction accuracy in the top_1 classification. Moreover, by adjusting the value of *k* in the Top_k classification, our methodology provides more control to its users and more choices to select optimal security versus classification accuracy based on the AI application design, as described in depth in Section 2.3.

#### 2.2.1. Category of Image Dataset

Under adversarial machine learning (AML), we run each adversarial attack individually on the DNN model, ResNet-34, using the test set in the CIFAR-100 dataset, which contains 10K images. The attack success ratio of each of the adversarial attacks varies, as illustrated in Table 1. During the categorization process, as represented in Algorithm 1, each image (x) is first checked by the DNN model for the correct label. The mispredicted result from DNN(x) adds x directly to the SETmis set. In contrast, the correct prediction of x passes to the AML(x) algorithm for a trial (e.g., FGSM), and the successfully applied attack output is added to the SETadv set. The unsuccessful attack moves x to the SETcrc set. In summary, we categorize the outputs into three sets as follows:

- SETcrc: The set of images that the DNN can correctly identify;
- SETmis: The set of images that the DNN misidentifies (misclassification);
- SETadv: The set of images produced by AML that can successfully and deliberately make the DNN misidentify as another object the attacker wants.

The percentage of misclassified images (SETmis) is maintained at 22.54 across various attacks. However, the percentages for the other categories vary depending on the strength of each attack and its parameters. Generally, the adversarial generated samples (SETadv) or the attack success ratio receive the highest percentage among other sets in all four adversarial attacks.
**Algorithm 1:** Categorize Image Dataset.**[SETcrc, SETmis, SETadv] = Category (Image dataset, DNN classification results)****Input:**{x,rightlabel}∈CIFAR-100(testset),DNNmodelDNN(x),adv_attackAML(x)**Output:** The three categories of image dataset according to DNN model classification and AML results.Initialize SETcrc, SETmis, SETadv to be all empty**for** image x∈ CIFAR-100 **do**    **if** DNN(x) is mispredict **then**        SETmis ← x    **else**        **if** DNN(x) is correct and AML(x) fail **then**           SETcrc ← x        **else**           SETadv ← x        **end if**    **end if****end for****return** [SETcrc, SETmis, SETadv]

#### 2.2.2. Detection Algorithm

The adversarial image detection model, denoted as Adv−aware(x), is addressed in Algorithm 2. We first pass a test image (*x*) to the primary DNN model, which is DNN(x) with Top_1, and to the secondary model, which is the RF model with Top_k donated, as denoted by RF(x, k). Then, we have two outputs: a single-class prediction from the primary DNN model (*y*) and *k* class predictions from the secondary model (Top_k) as a list of *k* classes. We check whether *y* predicted classes exist in the Top_k prediction list. If *y* exists in the Top_k, then it returns a boolean “false” value for forged status with the DNN(x) label (*y*). Otherwise, it returns “true” without a label or none, which detects a possible adversarial sample.

For instance, as shown in Figure 1, we use a *STOP* road sign as an input sample to our model. It passes to the primary model and the secondary model concurrently. In an adversarial attack scenario where the *STOP* sign image is a manipulated image, the predicted class from the primary model is *SPEED LIMIT 70*, whereas the second model provides a Top_3 list of predictions, for example, *STOP*, *Roundabout*, and *No entry*. Our model detects the input image as a forged “true”, since the predicted class from the primary model does not exist in the list of the secondary model. In the case of clean detection, the predictions have to be found in both model predictions. During our evaluation, we excluded misclassification samples in this section, which are tackled in Section 2.3.
**Algorithm 2:** Adversarial-Aware Deep Learning System.**[forged, label] = Adv-aware (x)****Input:** image x.**Output:** Whether the image is forged by adversarial attack or a clean image; classification label if x is a clean image.*y*← DNN(x) # DNN model classification label for the image xTop_k ← RF(x, k) # The top k group of labels generated by the RF classification model**if** *y*∈Top_k **then**    forged = false; label = *y***else**    forged = true; label = None**end if****return** [forged, label]

### 2.3. Defense System Adaptive Design

This section discusses a new technique for selecting the best value of *k* in the Top_k used in the secondary model based on the underlying application-specific requirements in terms of accuracy and security. Some applications require zero tolerance for attack success. On the other hand, a low success ratio of adversarial attacks in some other applications does not cause severe damage. Moreover, including the misclassification samples in this adaptive design improves the overall detection accuracy of adversarial attacks. The details are explained in the following subsections.

#### 2.3.1. Outputs of Our Proposed Adversarial-Aware Image Recognition System

Our image recognition system has two possible outputs: (1) the image under inspection is authentic, and its *identified* label is provided, or (2) the image under inspection is forged by AML and tagged as *forged*. Therefore, given that there are three possible sets of images in terms of DNN identification (introduced in Section 2.2.1), here are the six possible decision scenarios for our proposed system:

- Decision A ($Dec_{a}$): An image in SETcrc that is authentic and correctly identified;
- Decision B ($Dec_{b}$): An image in SETmis that is correctly identified as forged;
- Decision C ($Dec_{c}$): An image in SETadv that is correctly identified as forged;
- Decision D ($Dec_{d}$): An image in SETcrc that is misidentified as forged;
- Decision E ($Dec_{e}$): An image in SETmis that is misidentified as authentic and misclassified;
- Decision F ($Dec_{f}$): An image in SETadv that is misidentified as authentic.

From a user’s perspective, $Dec_{a}$, $Dec_{b}$, and $Dec_{c}$ are all ‘good’ and rightful decisions, whereas $Dec_{d}$, $Dec_{e}$, and $Dec_{f}$ are wrongful decisions that could cause a negative impact/cost to the user.

#### 2.3.2. Adjustable Parameter in Our Proposed System

In our proposed adversarial-aware image recognition system, one critical parameter that can be adjusted/controlled by the end user is the value of *k* in the Top_k classification by the secondary model. It can be used to make a delicate tradeoff between increasing the defense accuracy of adversarial attack images and increasing the correct recognition of normal images. The secondary verification of the ML module determines if an image under inspection belongs to one of the Top_k classes among all possible classification classes. Its classification setting (Top_k) can be, for example, Top_1, Top_10, Top_20, etc. When *k* increases, the classification decision by the DNN module has a higher probability of being included in the Top_k classes of the secondary verification system, which increases the possibility of good $Dec_{a}$ and the possibility of bad decisions ($Dec_{e}$ and $Dec_{f}$) as well.

In this paper, we present a solution to the above dilemma by translating and quantifying the problem into the optimization of a carefully defined objective cost function. We explain it in detail below.

#### 2.3.3. Using Objective Cost Function to Achieve Optimal Defense

Generally speaking, in most computer vision applications, a successful AML attack causes much more damage to the user than a misclassified event. In most cases, misclassifying an object/content in an image leads to a clearly identifiable wrongful conclusion, such that the user can easily know that it is a wrong identification, for example, misidentifying a road STOP sign as a red balloon in autonomous vehicle driving indicates that this is wrong image identification. However, a successful AML attack could make the user misidentify the STOP sign as a SPEED LIMIT sign, which could result in a serious car accident.

For this reason, when we decide how to adjust detection and defense settings for our proposed system, we should not use the classification accuracy, AUC score, or attack success rate directly as the metric. Instead, we define an overall cost objective function, that is, the weighted summation of all image classification results, to find the optimal defense parameters that minimize this objective function.

For the six decision outputs of our proposed system ($Dec_{a}$ to $Dec_{f}$), each decision for one image has its own cost (due to misidentification) or gain (due to correct identification), which can be treated as a positive or a negative cost. Let us define $C_{a}$, $C_{b}$, and $C_{c}$ as the gains for each of those three good decisions ($Dec_{a},Dec_{b}$, and $Dec_{c}$) and $C_{d}$, $C_{e}$, and $C_{f}$ as the cost values for each of those three wrongful decisions ($Dec_{d},Dec_{e}$, and $Dec_{f}$).

The objective cost function (Objf(k)) for choosing the optimal defense parameter (Top_k) in the secondary RF classification module is illustrated in Algorithm 3 and shown in Equation (1). We find the optimal value of *k* by selecting the minimum output ($min_{k}$) from the equation when changing *k* from 1 to 100. Parameters $N_{a}$ to $N_{f}$ refer to the number of times when decisions $Dec_{a}$ to $Dec_{f}$ happen, respectively.

(1) $$Objf\left(k\right)=min_{k}\left(C_{d}\cdot N_{d}+C_{e}\cdot N_{e}+C_{f}\cdot N_{f}-C_{a}\cdot N_{a}-C_{b}\cdot N_{b}-C_{c}\cdot N_{c}\right)$$

To calculate $N_{a}$, $N_{b}$, …, and $N_{f}$, a loop is conducted over the entire test set of the CIFAR-100 dataset. In Algorithm 3, each image (x) from the dataset is previously divided into three sets by Algorithm 1 (SETcrc, SETmis, and SETadv). Each if statement checks whether x image belongs to one of the sets and whether the outcomes of each model prediction (DNN and RF) are matched. For example, suppose x is a human object and DNN identifies it correctly, and the prediction also exists in the Top_3 RF outcomes. In that case, the decision state is set to $Dec_{a}$ and $N_{a}$ counter increments by one.

This optimization is conducted after the training stage, when we know the ground truth of all images, as shown in Section 3, and can calculate the values of $N_{a}$ to $N_{f}$ for each Top_k parameter for all test images. Since the number of possible values of *k* is limited (in our model, it has 100 possible values ranging from 1 to 100), there is no technical challenge in solving the optimization problem.
**Algorithm 3:** Adaptive Design Algorithm.**[k] = Adaptive(DNN classification results, RF classification results)****Input:** CIFAR-100(test set), DNN, RF**Output:** optimal parameter *k* for the secondary RF model**for** k∈{1,100} **do**    Set all the counters $N_{a},N_{b},\ldots ,N_{f}$ to 0    **for** image *x*∈ CIFAR-100 **do**        **if** x∈SETcrc&DNN(x)∈RF(x,k) **then**           $N_{a}++$        **else**           $N_{d}++$        **end if**        **if** x∈SETmis&DNN(x)∉RF(x,k) **then**           $N_{b}++$        **else**           $N_{e}++$        **end if**        **if** x∈SETadv&DNN(x)∉RF(x,k) **then**           $N_{c}++$        **else**           $N_{f}++$        **end if**    **end for**    Objective function $f\left(k\right)=\left(C_{d}\cdot N_{d}+C_{e}\cdot N_{e}+C_{f}\cdot N_{f}-C_{a}\cdot N_{a}-C_{b}\cdot N_{b}-C_{c}\cdot N_{c}\right)$**end for**Among all f(k),k∈{1,100} find the minimum $f(k^{*})$**Return** the optimal index $k^{*}$

#### 2.3.4. Examples of Adjusting Weights on Different Applications

In this section, we use several application scenarios to show why they need different cost weights in our adaptive design and the above optimization Equation (1). In different image classification applications, users can define the concrete values for the other cost factors according to their expert opinion and application scenarios. Four applications are introduced in the following, and Table 2 in the following section presents the outcomes of this adaptive method.

- **Autonomous driving**: We can define $C_{a}$ = 0.3, $C_{b}$ = 0.1, and $C_{c}$ = 0.5. The value of $C_{c}$ is higher than $C_{a}$ because in autonomous driving, it is more important for us to detect an adversarial attack than to correctly identify a normal roadside sign image. Similarly, we can define $C_{d}$ = 0.1, $C_{e}$ = 0.3, and $C_{f}$ = 0.8. We define $C_{f}$ as having a significantly higher value than others because $Dec_{f}$ means autonomous driving is compromised under a deliberate adversarial attack. For example, we could treat a STOP sign image as a right-turn-only sign, which could result in serious accident consequences. The value of $C_{e}$ is higher than $C_{b}$ in detecting misclassified images by the model due to the risk value we assume.
- **Healthcare**: Although deep-learning-based healthcare systems could achieve high accuracy in disease diagnosis, few such systems have been deployed in highly automated disease screening settings due to a lack of trust. Therefore, the human-based double-check process is usually used, and hence, the deep learning healthcare system can be tolerated in the security. Example values of the weights are $C_{a}$ = 0.7, $C_{b}$ = 0.4, $C_{c}$ = 0.1, $C_{d}$ = 0.4, $C_{e}$ = 0.1, and $C_{f}$ = 0.3. $C_{a}$ is the highest cost weight because the physician will most likely discover failure in other decisions during manual double checking.
- **Face recognition in checking work attendance**: Misrecognition or adversarial impact is low because the potential of utilizing these challenges by the employees is rare. Therefore, we can obtain higher positive gain values with $C_{a}$ = 0.7, $C_{b}$ = 0.4, and $C_{c}$ = 0.2. In contrast, we can value the negative decisions as $C_{d}$ = 0.4, $C_{e}$ = 0.2, and $C_{f}$ = 0.2.
- **Detecting inappropriate digital content**: Mispredicting nudity images to protect children is another example where the costs of an AML attack are medium—not as risky as in autonomous driving, nor as tolerable as in face recognition. Hence, we can choose $C_{a}$ = 0.7, $C_{b}$ = 0.1, $C_{c}$ = 0.2, $C_{d}$ = 0.3, $C_{e}$ = 0.1, and $C_{f}$ = 0.1.

#### 2.3.5. The Cost of Misclassified Clean Images

As of today, there are no image classification models that can provide a 100 percent accurate result. Table 1 shows the accuracy rates of various DNN models without any attacks. ResNet-34 achieves an accuracy rate of 77.47 percent, while VGG16 has a lower accuracy rate of 72.25 percent. On the other hand, DenseNet boasts a higher accuracy rate of 78.69 percent. The percentage of misclassified images is enormous. Therefore, the business models of AI applications should consider these failure cases to assess their risks in case of using any DNN model with a high percentage of misclassification. On the other hand, our approach can detect a significant fraction of these detection failures and categorize them as forged by adversarial attacks.

As described in the previous section, $Dec_{b}$ can identify the misclassification of tested samples and be counted as positive to DNN model accuracy. On the other hand, $Dec_{e}$, where our approach wrongly identifies it as forged, is counted as negative to the overall accuracy. Application designers can define the costs of these decisions, balancing security and safety with passing tolerance using Algorithm 3. The accuracy of the overall system can be significantly affected, as demonstrated in the following section.

#### 2.3.6. Evaluation Metric

The evaluation technique for our proposed method is similar to those presented in previous works on detection methods [10,11,12]. We use the area under the ROC curve (AUC) score in our assessments between clean ($Dec_{a}$) and adversarial images ($Dec_{c}$), as addressed in Section 3. Accuracy (acc.) is another metric used to evaluate our proposed model based on image classification application parameters introduced in Section 2.3 and Section 3.

## 3. Results

In this section, we showcase the evaluation and outcomes of our study. First, the settings for the experiments and the utilized environment are explained. Then, the configurations for the adversarial attacks we deploy to target the various deep learning models are outlined. Lastly, we present and compare the main results according to each proposed approach in Section 2.2 and Section 2.3.

### 3.1. Experimental Setup

To evaluate the robustness and effectiveness of the proposed scheme, we run our training, evaluation, and attacks using an NVIDIA GeForce RTX 3090 GPU. We use the Sklearn [37] open-source Python library for the classical ML random forest model. On the other hand, we use PyTorch-lightning [38] for DNN models. Finally, we use Torchattacks [39] to run the adversarial attacks.

### 3.2. Adversarial Attack Configuration

The attacker knows that the targeted image classification system uses ResNet-34 to train the image classification model. He/she also knows the data being used for that training, i.e., the CIFAR-100 training set. The attacker uses a test set of the same dataset and state-of-art adversarial attack algorithms: FGSM [1], Deepfool [13], CW [14], and PGD [15]. The parameters of each type of AML are listed in Table 3 and defined in the next section.

In the FGSM trial, we set the ϵ parameter, which is a hyperparameter determining the size of the perturbations introduced to the input data, to 0.007. The value of ϵ is a tradeoff between the adversarial attack strength and the perturbation perceptibility. Raising this value could increase the exploit success rate; however, it might show apparent noise on the targeted image that could be revealed to human perception. We set the default value to 0.007 because the added perturbations are not easily perceived by human eyes. The FGSM attack success accuracy based on the selected ϵ on the CIFAR-100 test set is 65.75%.

To execute the Deepfool attack, we limit the attack iterations to 50 steps before stopping. During each iteration, the attack calculates the direction of the closest decision boundary to the original input data point in order to determine the minimum perturbation required to deceive the targeted DNN model. The overshoot parameter is set to 0.02, which multiplies the computed perturbation vector and adds it to the input image. With these settings, the attack success accuracy reaches 99.92%.

To ensure a successful attack by the CW method, we utilize the C&W attack parameters listed in Table 3: c=1, κ = 0, steps s=50, and lr = 0.01. The ‘*c*’ hyperparameter determines the magnitude of the perturbation, while the margin parameter (κ) determines the confidence gap between the predicted and target classes. The steps (*s*) parameter represents the number of iterations required for the attack to succeed or end. Lastly, the learning rate (lr) controls the optimization iteration steps. With these adjustments, we achieve an attack success rate of 98.64%.

The PGD attack is adjusted with the following parameters: ϵ = 0.03, alpha α = 0.004, and steps = 40. ϵ; steps were explained in previous attacks, while alpha functions similar to the learning rate determine the size of each optimization step. This attack has a success rate of 98.83%.

### 3.3. Main Results

Table 4 summarizes the AUC scores of four adversarial attack detectors with our proposed method from Section 2.2 using features from all the DNN penultimate layers. For comparison, we compare our proposed method with four other popular adversarial detection methods: DkNN [9], LID [10], Mahalanibis [11], and NNIF [12].

Overall, our proposed Top_1 threshold surpasses other methods in most attacks, as indicated in bold, while the LID method is the least effective in detecting attacks. The best FGSM attack detection corresponds to our proposed Top_22 method. Additionally, the NNIF model is the second-best detector approach to resist all attacks. The AUC scores at FGSM show a roughly 10 percent gap between the detectors. In contrast, in Deepfool, the gap is much more pronounced, with LID scoring 52.25 and our proposed Top_1 scoring 97.57.

**Figure 3:**
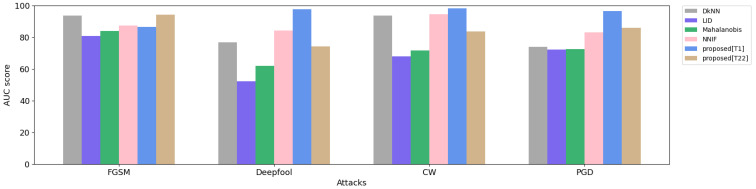
AUC score comparison for adversarial attack detectors. The x-axis represents the detector methods. The y-axis represents the AUC score of adversarial detectors. Each color demonstrates one of the detectors, as listed in the top-right legend.

The AUC score comparisons for different adversarial detector models on various attacks are shown in Figure 3. The x-axis represents the four adversarial attacks, while the y-axis describes the AUC score, ranging from 0 to 100. Each color on the graph represents one defense method, as represented in the top-right legend, namely DkNN, LID, Mahalanibis, NNIF, proposed[T1], and proposed[T22] represented by gray, navy, light green, light pink, light blue, and light brown, respectively. In the FGSM attack, DkNN and the proposed[T22] method were the most effective defense mechanisms, while the others showed slight differences, with a score of around 80. The Deepfool bars show significant improvement in detection methods, but some methods have noticeable weaknesses. For the CW attack detection, DkNN, NNIF, and the proposed[T1] perform well, while the others score an average of 70. Finally, the PGD attack is proven to be powerful against DkNN, LID, and Mahalanibis, with a semi-matching AUC score of 72, while the remaining methods show significant improvement, with the proposed[T1] method scoring the highest, with a score of 96.

For our proposed system, there is an inherent tradeoff between higher accuracy in detecting adversarial attacks and higher classification accuracy for clean data inputs, as illustrated in Table 2. We assign varying weights to each application depending on the potential risks we might face in the event of overlooking a successful attack and depending on our preferred accuracy in classifying normal clean inputs. Our adaptive optimization algorithm (Equation (1)) determines that the optimal settings for the RF Top_*x* probability index should be as follows. In all types of attacks, autonomous driving takes the Top_1 due to the potential for severe accidents if adversarial or misclassified samples are not detected. In health care, FGSM takes Top_5, and the remaining attacks all take Top_1. A face recognition application selects Top_3 for FGSM and Top_1 for the rest. Finally, detecting inappropriate content on a system selects Top_14 for the FGSM attack and Top_1 for the other attacks.

In Section 2.3.5, we discussed how misclassification samples could improve the accuracy of the ResNet-34 model in detecting adversarial attacks. To demonstrate this, we conduct an FGSM attack experiment using the same applications and weights as in Table 2. We present the results in Table 5. First, we calculate the accuracy without the misclassification samples using Equation (1). Then, we calculate the accuracy again after including misclassification samples ($C_{b}\cdot N_{b}$ and $C_{e}\cdot N_{e}$), as displayed in Table 5. Our approach effectively enhances the AML detection accuracy on the ResNet-34 model, which was initially predicted with 74.47 percent accuracy.

## 4. Discussion

This section links our proposed ideas with the results and provides a more insightful summary and discussion. We begin by justifying the models and the obtained results. Following this, we elaborate on our model analysis, utilizing a high-accuracy DNN model. Next, we present the challenges associated with this research. Lastly, we introduce our future plans for this project.

### 4.1. Justifications

Although the RF is a sufficient model in regression [30] and multiclassification applications [40], it is not commonly used for image classifications because images have a large number of pixels, resulting in high-dimensional feature spaces. In addition, image processing is computationally expensive and time-consuming during training. However, we decided to use RF as a secondary model for two reasons. Firstly, other models such as support vector machine (SVM) [41] are not efficient in multiclassifications and are computationally expensive. Secondly, we want to showcase the usefulness of having two different architectural models to overcome adversarial attacks. Studies such as [9,12] have used traditional ML algorithms to create AML detectors. They adapted *k*-NN in generating their adversarial detectors by adding a *k*-NN model between DNN layers during training to extract new features that can be analyzed and used to recognize clean and noisy samples versus adversarial ones. However, in addition to *k*-NN’s extreme complexity and high computational performance, these studies found that different types of attacks have varying resistances depending on the effectiveness of the attack in generating perturbations to fool the model.

For example, Table 4 shows fluctuations in AUC scores in resisting each attack by every detection algorithm, such as NNIF for FGSM, Deepfool, CW, and PGD, with scores of 87.23, 84.20, 94.58, and 83.09, respectively. In contrast, our proposed system with the Top_1 setting is consistently effective, regardless of FGSM outcomes, as it has a large number of clear samples that are not affected by the attack at α 0.007; further clarification is provided later in this section. Therefore, a new technique of attack that relies on backpropagation could harden the defense algorithms, as illustrated in [12], when a detector trained on an FGSM attack is only tested on unseen attacks such as Deepfool. These findings indicate a decrease in performance when testing for unseen attacks compared to seen attacks. Our proposed system, however, is tested on all adversarial attacks without attack pattern evaluation nor DNN model changing and presents a generalization across different attacks.

Additionally, the results of the FGSM attack in Table 2 show reasonable changes with Top_*k* based on an application’s weight parameters. This change from Top_1 in autonomous driving to Top_14 in detecting inappropriate content is normal when we increase the cost of $C_{f}$. In this situation, Equation (1) significantly increases the security sensor to minimize the success rate of adversarial attacks. In contrast, the equation reduces the model sensitivity when preventing inappropriate content because the risk of successful attacks is not so serious. This equation provides freedom to the application developer to choose the best and most optimal defense setup.

Unlike the other attacks, Table 2 shows that we consistently use Top_1 for every test of Deepfool, CW, and PGD attacks because of a couple of reasons. First, the variation in the success rate of these attacks, as shown in Table 3, is based on the attack strategy and the strength to fool the model. For instance, PGD is developed from an FGSM attack, where PGD has a selected number of iterations to break the model, while FGSM applies one-time manipulation based on the ϵ value. Additionally, the success ratio is exemplary or unrealistic. In real-world attacks, attackers have no access to information about the ML models, the data used for training, the integrated security level, etc. The regular success rate should be much less than that in the examples presented in the table. An FGSM attack is an example of the successful usage of our proposed adaptive design theory; otherwise, the system’s adaptivity is useless if the applied systems are extremely exposed to adversarial attacks.

### 4.2. Model Scalability

Our experimental construction is based on the ResNet-34 model, which has an accuracy of 77.47 percent. We select this model for our demonstration to match previous experimental setups and compare our output enhancement. To elaborate and to present our approach efficiently, we train the ViT-B_16 model on the CIFAR-100 dataset and achieve 92.58 percent accuracy. Then, we attack the model with an FGSM attack. Our defense shows validity in detecting adversarial attacks, for which we obtain 78.90 with Top_1 and 89.49 with top_40 based on the AUC score. Comparing ResNet-34 with ViT-B_16, our approach selects smaller k on ResNet-34 for the following reasons. First, the accuracy of ViT-B_16 before the attack is higher by 14 percent. Next, the overall accuracy of random forest is low compared to DNN models, which makes k changes regarding the primary model accuracy. Finally, the classical model accuracy is careless since the DNN model is the main model the application relies on for classification, and we adapt the idea of k to overcome this challenge.

### 4.3. Challenges

Challenges are found throughout this study. Foremost, the RF model is designed professionally for structural and tabular applications such as stock market price predictions that use a specific number of vectors; simple image classification; or recognition tasks, such as satellite imagery object detection. However, the RF model capability can be limited when faced with large-scale training involving a significant number of classes, such as when using the ImageNet dataset [42] with its 1000 classes and 1.2 million images. To tackle these challenges, a DNN model was developed. It excels in extracting features from high-dimensional vector spaces and large datasets while requiring less time for training. We use this RF model as a prototype for our analysis, and we highlight that a potential robust defense mechanism exists if we can adopt a different architecture.

### 4.4. Future Work

In our future work, we aim to improve our detector by dealing with the scalability issue in classical ML models. To achieve this, we plan to use feature vectors from DNN fully connected layers and input the outputs to RF. This strategy will create a more effective detector using RF as a classifier and DNN as a feature extractor. By combining the strengths of both models, we can benefit from DNN’s superior feature extraction and RF’s outstanding ability to mitigate adversarial attacks. In addition, our proposed system has a high chance of success, even if the attacker is familiar with the random forest method discussed here because most adversarial attacks in computer vision rely on the gradient, which is a primary function in DNN models but is not utilized in the random forest model. However, we should study how attackers can potentially bypass our defense measures. This includes testing if an attacker can deceive our detection methods while being aware of the second approach used to identify adversarial attacks.

## 5. Conclusions

Our paper presents a straightforward yet impactful approach to identifying adversarial attacks. It involves utilizing a secondary classical machine learning model in conjunction with the primary DNN model for image classification. The secondary model’s architecture completely differs from the primary model to thwart adversarial attacks that rely on the backpropagation technique, which generates an effective perturbation targeting the primary deep learning model. Our proposed detector outperforms state-of-the-art models that rely on analyzing adversarial sample behavior and patterns during DNN training. Additionally, our model requires no modifications to the DNN model or learning attack types. We use the CIFAR-100 dataset for this study, as it contains a reasonable number of classes to fulfill this task, considering all the challenges with the RF model in image classifications tasks.

## Figures and Tables

**Table 1:** Accuracy comparison of different DNN models before and after adversarial attacks on the CIFAR-100 dataset.

	Attack	Targeted Model	Untargeted Models	
		ResNet-34	VGG16	DenseNet
**Accuracies (%)**	Without attack	77.47	72.25	78.69
	FGSM	34.25	35.09	36.19
	Deepfool	25.78	24.79	24.84
	CW	25.77	24.49	25.0
	PGD	22.58	22.87	22.7

**Table 2:** AUC score comparison based on different application preferences.

Application	Wight	Accuracy Based on Best Top_n Selection from Formula (1)							
		FGSM	acc.	DeepFool	acc.	CW	acc.	PGD	acc.
**Autonomous driving**	$C_{a}=0.3$ $C_{b}=0.1$ $C_{c}=0.5$ $C_{d}=0.1$ $C_{e}=0.3$ $C_{f}=0.8$	Top_1	81.14%	Top_1	89.84%	Top_1	89.68%	Top_1	90.04%
**Health care**	$C_{a}=0.7$ $C_{b}=0.4$ $C_{c}=0.1$ $C_{d}=0.4$ $C_{e}=0.1$ $C_{f}=0.3$	Top_5	77.66%	Top_1	89.84%	Top_1	89.68%	Top_1	90.04%
**Face recognition**	$C_{a}=0.7$ $C_{b}=0.4$ $C_{c}=0.2$ $C_{d}=0.4$ $C_{e}=0.2$ $C_{f}=0.2$	Top_3	79.06%	Top_1	89.84%	Top_1	89.68%	Top_1	90.04%
**Inappropriate content**	$C_{a}=0.7$ $C_{b}=0.1$ $C_{c}=0.2$ $C_{d}=0.3$ $C_{e}=0.1$ $C_{f}=0.1$	Top_14	70.14%	Top_1	89.84%	Top_1	89.68%	Top_1	90.04%

**Table 3:** Experiment settings.

Targeted Model	Dataset	Adversarial Attack	Parameters	Attack Success Ratio (%)
ResNet-34	CIFAR-100	FGSM	ϵ = 0.007	65.75
		Deepfool	s = 50, overshoot = 0.02	99.92
		CW	c = 1.0, κ = 0, s = 50, lr = 0.01	98.64
		PGD	ϵ = 0.03, α = 0.004, s = 40	98.83

**Table 4:** AUC score of adversarial detection methods.

Detector	AUC Score			
	FGSM	Deepfool	CW	PGD
DkNN [9]	93.65	76.71	93.77	73.78
LID [10]	80.68	52.25	67.84	72.25
Mahalanibis [11]	83.90	62.05	71.60	72.46
NNIF [12]	87.23	84.20	94.58	83.09
Top_1	86.62	**97.57**	**98.21**	**96.49**
Top_22	**94.17**	74.17	83.50	86.04

**Table 5:** AML detection accuracy comparison before and after including misclassification samples.

Application	w/o Misclassification (%)	With Misclassification (%)
Autonomous driving	62.81	81.14
Health care	63.20	77.66
Face recognition	61.66	79.60
Detecting inappropriate content	60.08	70.14

## Abbreviations

The following abbreviations are used in this manuscript:

DNN	Deep neural network
ML	Machine learning
Top_k	The index of the top k classes of a model prediction
AML	Adversarial machine learning
AI	Artificial intelligence
FGSM	Fast gradient sign method
CW	Carlini and Wagner attack
PGD	Projected gradient descent attack
ComCNN	Compression convolutional neural network
k-NN	K-nearest neighbors
LID	Local intrinsic dimensionality
MART	Misclassification-Aware adveRsarial Training
AUC	Area under the ROC curve
SVM	Support vector machine
